# Interrogation of transcriptomic changes associated with drug-induced hepatic sinusoidal dilatation in colorectal cancer

**DOI:** 10.1371/journal.pone.0198099

**Published:** 2018-06-07

**Authors:** Monika A. Jarzabek, William R. Proctor, Jennifer Vogt, Rupal Desai, Patrick Dicker, Gary Cain, Rajiv Raja, Jens Brodbeck, Dale Stevens, Eric P. van der Stok, John W. M. Martens, Cornelis Verhoef, Priti S. Hegde, Annette T. Byrne, Jacqueline M. Tarrant

**Affiliations:** 1 Department of Physiology and Medical Physics, Royal College of Surgeons in Ireland, Dublin, Ireland; 2 Department of Safety Assessment, Genentech Inc., South San Francisco, California, United States of America; 3 Department of Oncology Biomarker Development, Genentech Inc., South San Francisco, California, United States of America; 4 Department of Epidemiology and Public Health Medicine, Royal College of Surgeons in Ireland, Dublin, Ireland; 5 Department of Surgical Oncology, Erasmus MC, Rotterdam, Netherlands; 6 Department of Medical Oncology, Erasmus MC, Rotterdam, Netherlands; University of Navarra School of Medicine and Center for Applied Medical Research (CIMA), SPAIN

## Abstract

Drug-related sinusoidal dilatation (SD) is a common form of hepatotoxicity associated with oxaliplatin-based chemotherapy used prior to resection of colorectal liver metastases (CRLM). Recently, hepatic SD has also been associated with anti-delta like 4 (DLL4) cancer therapies targeting the NOTCH pathway. To investigate the hypothesis that NOTCH signaling plays an important role in drug-induced SD, gene expression changes were examined in livers from anti-DLL4 and oxaliplatin-induced SD in non-human primate (NHP) and patients, respectively. Putative mechanistic biomarkers of bevacizumab (bev)-mediated protection against oxaliplatin-induced SD were also investigated. RNA was extracted from whole liver sections or centrilobular regions by laser-capture microdissection (LCM) obtained from NHP administered anti-DLL4 fragment antigen-binding (F(ab’)_2_ or patients with CRLM receiving oxaliplatin-based chemotherapy with or without bev. mRNA expression was quantified using high-throughput real-time quantitative PCR. Significance analysis was used to identify genes with differential expression patterns (false discovery rate (FDR) < 0.05). Eleven (CCL2, CCND1, EFNB2, ERG, ICAM1, IL16, LFNG, NOTCH1, NOTCH4, PRDX1, and TGFB1) and six (CDH5, EFNB2, HES1, IL16, MIK67, HES1 and VWF) candidate genes were differentially expressed in the liver of anti-DLL4- and oxaliplatin-induced SD, respectively. Addition of bev to oxaliplatin-based chemotherapy resulted in differential changes in hepatic CDH5, HEY1, IL16, JAG1, MMP9, NOTCH4 and TIMP1 expression. This work implicates NOTCH and IL16 pathways in the pathogenesis of drug-induced SD and further explains the hepato-protective effect of bev in oxaliplatin-induced SD observed in CRLM patients.

## Introduction

SD is an expansion of centrilobular hepatic sinusoids that can progress to sinusoidal obstructive syndrome (veno-occlusive disease (SOS/VOD)), a potentially life-threatening complication, which may also include peri-sinusoidal fibrosis and nodular regenerative hyperplasia. Sinusoidal dilation may be clinically silent [1] with diagnosis requiring at least two symptoms of jaundice, tender hepatomegaly and right-upper quadrant pain, ascites and/or unexplained weight gain [2]. SOS/VOD was responsible for the U.S. market withdrawal of gemtuzumab ozogamicin (Mylotarg®), an antibody-drug-conjugate of anti-CD33 and a calicheamicins cytotoxin for the treatment of patients with acute myeloid leukemia [3]. The presence of SOS/VOD in patients treated by partial hepatectomy for CRLM has been linked to higher risk of morbitidity, including increased peri-operative risk of bleeding, post-hepatectomy liver failure, lower long-term disease specific survival, intra-hepatic tumor recurrence and death due to multi-organ failure [4]. As yet, there are no accurate, non-invasive tests to confirm SOS/VOD diagnosis [2].

SOS/VOD is associated with exposure to pyrrolizidine alkaloid-containing herbal remedies [5], chemotherapy prior to hematopoietic stem cell transplantation [2] and oxaliplatin-based adjuvant or neo-adjuvant chemotherapy for CRLM [6, 7], DLL4 (an endothelium-specific NOTCH ligand) represents an attractive target in cancer therapy due to its role in tumor angiogenesis and its contribution to the maintenance and proliferation of cancer stem cells [8] with several anti-DLL4 antibodies having been developed and tested both in pre-clinical and clinical settings [9–11]. Unfortunately, pre-clinical studies in mice, rats and NHP have also reported anti-DLL4-associated hepatotoxicity characterized by SD and induction of vascular neoplasms in the liver [11, 12].

SD resulting from long-term exposure to anti-DLL4 suggests a role for DLL4/NOTCH signaling in maintaining the hepatic endothelium in a quiescent state. Moreover, it has been shown that chronic DLL4 blockade causes pathological activation of endothelial cells, disrupts normal organ homeostasis and induces vascular tumours raising important safety concerns [12]. Nevertheless, as yet there are no accurate, non-invasive tests to confirm SOS/VOD diagnosis [2]. Interestingly, while the molecular mechanisms underpinning hepatotoxicity associated with drug-induced SD and/or SOS/VOD remains unclear, clinical observations suggest that bev (a monoclonal humanized antibody against vascular endothelial growth factor (VEGF-A) has a protective effect against oxaliplatin-related sinusoidal damage in the pre-operative CRLM setting [7, 13]. However, the underlying mechanism of action has not been fully elucidated.

Herein, to interrogate the molecular pathogenesis of SD, we implemented a targeted transcriptomics approach to interrogate gene expression changes associated with drug-induced hepatic SD. We employed a robust anti-DLL4-induced NHP model that provided strong, direct evidence that blockade of DLL4/NOTCH1 signaling causes liver toxicity. Liver tissues from our NHP model treated with anti-DLL4 or CRLM patients treated with oxaliplatin were used to identify key genes involved in the pathogenesis of hepatic SD. We further investigated the protective effect of bev to counteract oxaliplatin-induced hepatic SD.

## Materials and methods

### Pre-clinical NHP study design

Twenty-four, 3–6 year old, naïve male and female cynomolgus monkeys (Chinese origin *Macaca fascicularis*) with weight range of 2.3–4.7 kg (mean weight of 3.4 kg and 3.0 kg, for males and females, respectively) sourced from Covance Research Products Inc. (Alice, Texas, US) were randomised using a computerized procedure designed to achieve body weight balance, into 4 terminal treatment groups (n = 3/sex/group). NHP were administered either vehicle (20mM sodium succinate, pH5.5; 240mM sucrose; and 0.02% polysorbate20) or anti-DLL4 F(ab’)_2_ formulated in vehicle diluent at concentrations 5, 15, or 50 mg/kg/week, by intravenous injection via the saphenous vein once weekly for 8 weeks (9 doses in total) at a dose volume of 5 mL/kg. Dosing occurred in the morning and was performed outside of the home cage. Dose levels were selected to approximate and provide multiples over the anticipated human clinical exposures. The intravenous route of administration was the intended clinical route. NHP procedures including blood collection and necropsy were performed in randomized approach (generated by a computer) although the staff were not blinded to the dose group allocation.

The NHP were individually housed in stainless steel cages (height of 89 cm, enclosure size of 0.42 m^2^ except during periods of commingling) and were offered Certified Primate Diet (PMI #5048) one to two times daily. Water was offered *ad libitum*. Environmental controls were set to maintain the following animal room conditions: temperature range of 20°C to 26°C, relative humidity range of 30% to 70%, 10 or greater air changes/hour, and a 12-hour light/12-hour dark cycle. The light/dark cycle was interrupted for study-related activities. Various cage-enrichment devices and fruits, vegetables, or dietary enrichment were provided. NHP were also commingled in accordance with standard operating procedures of the facility to provide psychological enrichment unless precluded for study procedures, behaviour, or health reasons. Veterinary care including medical treatment necessary to prevent unacceptable pain and suffering, including euthanasia was the responsibility of the attending laboratory animal veterinarian.

The total number of NHP used in this study, as well as the group sizes and number of groups, was the minimum number required to properly characterize the safety profile of the test article, which was the primary reason for conducting the study. This determination was based on previous regulatory experience for safety assessment studies in NHP submitted to health authorities and in adherence to the 3R’s principles to reduce and refine animal use.

The anti-DLL4 F(ab’)_2_ therapeutic binds and blocks human, NHP, and rodent DLL4. Pharmacologic activity of the parental anti-DLL4 mAb (YW152F) and F(ab’)_2_ have been characterized [14, 15].

At terminal necropsy, NHP were anesthetized with sodium pentobarbital, exsanguinated, and necropsied. Animals were fasted overnight prior to necropsy. Liver tissue was collected in 10% neutral buffered formalin and fixed samples were then embedded in paraffin, sectioned and stained with hematoxylin and eosin (H&E) for evaluation. Two pathologists conducted independent microscopic examination of the liver sections and findings were verified by consensus. Each SD lesion was assigned a severity score on a four-point [16].

The NHP study was conducted at Covance Laboratories (Madison, WI) under Good Laboratory Practices. All *in vivo* protocols were approved by the Covance Institutional Animal Care and Use Committee (11–684) and complied with their guidelines. All procedures were in compliance with the Animal Welfare Act, the Guide for the Care and Use of Laboratory Animals, and the Office of Laboratory Animal Welfare. Covance Laboratories holds an approved Animal Welfare Assurance in compliance with Public Health Service Policy on Humane Care and Use of Laboratory Animals (#A3218-01), is accredited with the Association for Assessment and Accreditation of Laboratory Animal Care (AAALAC) and is registered with the United States Department of Agriculture (USDA) #35-R-0030.

### Clinical study design

Liver samples were acquired from 65 patients, who had partial hepatic resection for CRLM between the years 2009 and 2012. Formalin-fixed, paraffin-embedded (FFPE) samples were sectioned and stained with H&E for routine histopatological review. Of the 65 consecutive cases, 40 received neo-adjuvant oxaliplatin-based chemotherapy and 25 received oxaliplatin and bev prior to liver resection. The median number of courses of oxaliplatin and bev was 4 and 3 respectively. Mcroscopic evaluation was conducted by a pathologist scoring the presence of SD in the non-tumoral liver distant from the peri-tumoral area. An internally consistent 4-point scale of SD extent was used [7]. Informed consent was obtained to use residual tissue for research. The data and tissue used in the current study was anonymized according to Good Clinical Practice Guidelines. As prescribed by national regulations, the current study was not subject to the “Medical Research Involving Human Subjects Act”. The institutional ethics committee, Medical Ethical Committee Erasmus Medical Centre, approved the study.

### LCM and processing of NHP tissue

Livers with SD present or absent were derived from NHPs treated with 15 mg/kg of anti-DLL4 or vehicle, respectively. The centrilobular hepatic zones were microdissected using a Leica LMD6000 laser microdissector (Leica Microsystems, Buffalo Grove, IL) (S1 Fig). The total volume of tissue dissected from each sample was 6.86±0.52 mm^3^ (approximately 50 dissected hepatic zones). Microdissected tissue was incubated overnight at 37°C with proteinase K solution (ARCTURUS® Paradise® PLUS WT-RT Kit, Life Technologies, Carlsbad, CA). RNA isolation, DNase treatment, and WT-RT were performed according to the manufacturer’s protocol. cDNA generated from RNA samples using random primers was then pre-amplified with a pool of TaqMan assays using the Invitrogen SuperScript® III Platinum® One-Step qRT-PCR Kit (Life Technologies, Carlsbad, CA) as per the manufacturer’s protocol. Alternatively, five FFPE whole liver sections with SD present or absent derived from anti-DLL4 or vehicle-treated NHPs had RNA extracted using the High Pure FFPE RNA Micro Kit (Roche Applied Sciences, Indianapolis, IN) according to the manufacturer’s protocol. All RNA samples were stored at -80°C. 100 ng/ul of RNA was pre-amplified with a pool of TaqMan gene expression assays pre-validated for NHP species and tissue specificity (S2 and S5 Figs) then stored at 4°C.

### Processing of clinical tissue

RNA was isolated from five FFPE whole liver sections per patient using the High Pure FFPE RNA Micro Kit (Roche Applied Sciences, Indianapolis, IN) according to the manufacturer’s protocol.

### High-throughput real-time quantitative PCR of NHP and clinical samples

Pre-amplified cDNA samples were diluted with TE buffer and amplified using Taqman Universal PCR Master Mix (Applied Biosystems, Foster City, CA) on the BioMark^TM^ HD system (Fluidigm, South San Francisco, CA) according to the manufacturer’s protocol (further details provided in S1 File). An average of the Ct values of the reference genes (ARF5, ARFGAP2, ARL1, SP2, TMEM55B and VPS33B for NHP samples and ACTB, ARF5, B2M and GAPDH for clinical samples) were calculated and expression levels of liver target genes were determined by -ΔCt = -(mean Ct(target gene)–mean Ct(reference genes)).

### Statistical analysis

Statistical analysis of gene expression levels in NHP and clinical liver samples was performed in the SAS/STAT software (SAS Institute Inc., Cary, NC, USA) using ANOVA/two sample t-test or two-way ANOVA respectively. P-values were corrected for multiple comparisons errors using the FDR (Benjamini-Hochberg) method [17]. Gene sets with FDR<0.25 [18], were used for hierarchical clustering and principal component analysis (PCA) that were performed in MultiExperiment Viewer software [19]. The FDR significance level was set at 0.05.

## Results

### Identification of differentially expressed genes in zonal regions of NHP livers with anti-DLL4-induced SD

**Fig 1:**
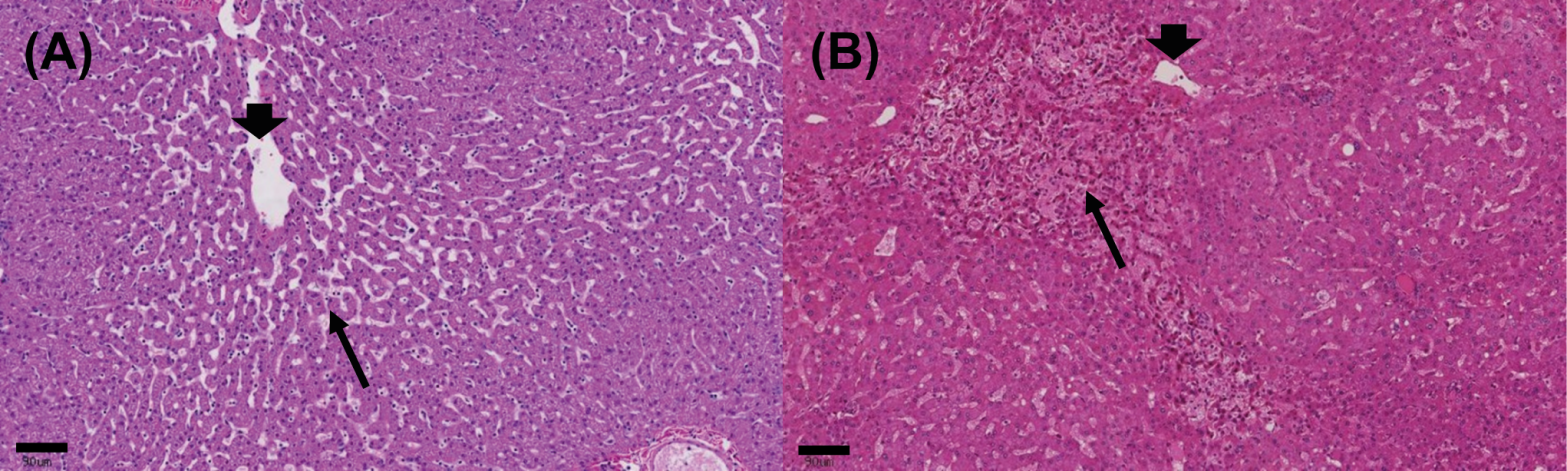
Liver sinusoidal dilation. **(A)** NHP administered 50 mg/kg anti-DLL4 and **(B)** CRLM patient treated with oxaliplatin both have sinusoidal dilatation in the centrilobular area near the central vein with a severity grade of 3. Thin arrows point to dilated sinusoids, and thick arrows to the central veins. The human liver **(B)** has blood retained within the dilated sinusoids that imparts a pink color. Scale bar = 90 μm.

**Fig 2:**
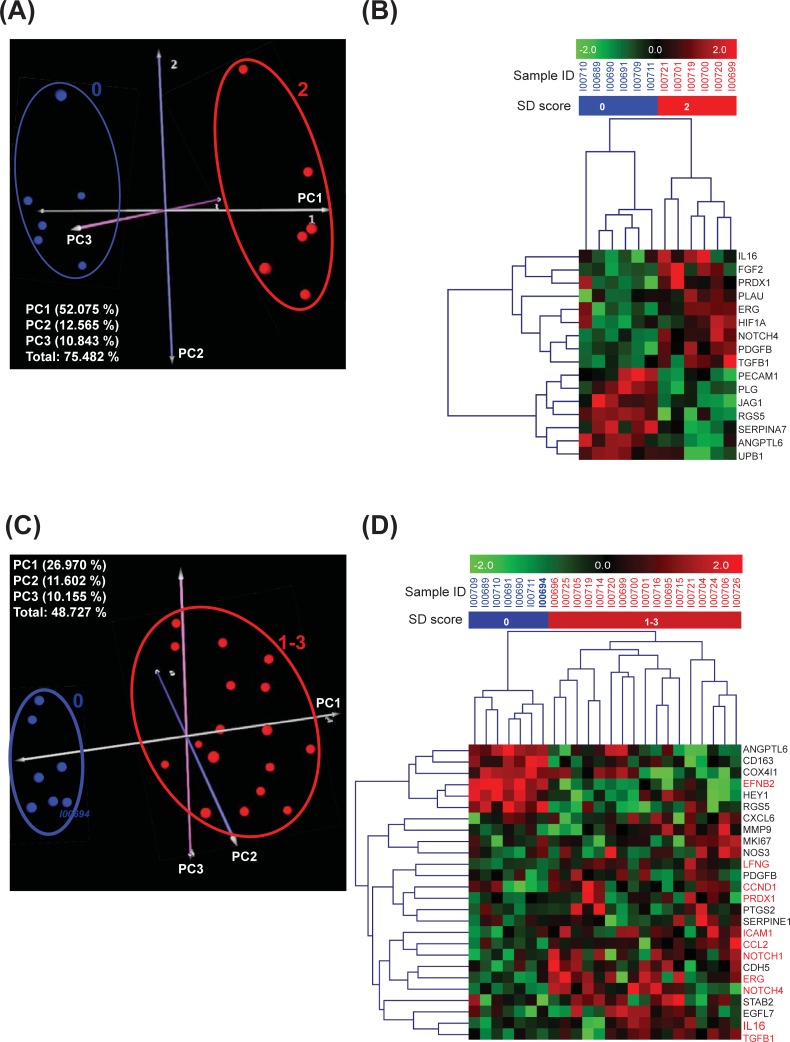
PCA and hierarchical clustering of NHP FFPE LCM hepatic regions and whole liver samples with or without anti-DLL4-associated SD based on their gene expression with FDR cut off at 0.25. Results from microfluidic high-throughput RT-qPCR were normalized by calculating–ΔCt using the average of reference genes. PCA **(A)** and hierarchical clustering based on Pearson correlation **(B)** were undertaken using genes with differential expression (FDR<0.25) between the LCM hepatic regions derived from liver samples of NHPs with SD present (moderate SD severity score 2 (anti-DLL4 15 mg/kg) n = 6)) and absent (SD severity score 0 (vehicle) (n = 6)) LCM hepatic regions. PCA **(C)** and hierarchical clustering based on Pearson correlation **(D)** were undertaken using genes with differential expression (FDR<0.25) between whole liver samples of NHPs with SD present (mild to severe SD severity scores 1–3 (anti-DLL4 5–50 mg/kg) (n = 17)) and absent (SD severity score 0 (vehicle or anti-DLL4 5 mg/kg) (n = 7)). PCA data are presented as spheres in PC1, PC2 and PC3 3 dimensional (3-D) space. The axes have been rotated to highlight the separation of the distinct clusters. The blue spheres surrounded with blue line represent the samples without SD and the red spheres surrounded with red line represent the samples with SD. The silver, blue, and pink axes represent principal components 1, 2 and 3 respectively **(A and C)**. In hierarchical clustering, each row represents a gene and each column represents a sample. Red squares indicate high gene expression; green squares indicate low gene expression **(B and D)**.

Treatment of NHP with anti-DLL4 F(ab’)_2_ reliably induced sinusoidal dilation in the centrilobular zone of the liver (Fig 1A) [12, 15, 16]. To identify differentially expressed genes present in centrilobular hepatic areas affected with SD, we dissected these regions using LCM (S1 Fig). NHP liver samples without SD (vehicle, SD severity score 0, n = 6) and with moderate SD (15 mg/kg anti-DLL4, SD severity score 2, n = 6) were used. Sixteen differentially expressed hepatic genes were identified with FDR<0.25, of which none reached significance (FDR<0.05) (Table 1). PCA on the identified gene-set (FDR<0.25) revealed the total contribution rate of the first 3 principal components reached 75.48% of the total data variability and partitioned samples into distinct domains based on presence of SD (Fig 2A). A set of 16 genes also separated liver samples into two clusters based on the presence of SD in hierarchical clustering (15 mg/kg anti-DLL4- or vehicle-treated) (Fig 2B).

**Table 1:** List of differentially expressed genes in LCM hepatic regions-derived from NHPs with moderate anti-DLL4-associated SD (severity score 2, SD 2) (n = 6) compared to LCM hepatic regions-derived from NHPs without anti-DLL4-associated SD (severity score 0, SD 0) (n = 6).

Gene symbol	Gene name	Direction	p-value (SD 2 vs. SD 0)	FDR (SD 2 vs. SD 0)
FGF2	Fibroblast growth factor 2	↑	0.0033	0.0651
NOTCH4	Notch 4	↑	0.0019	0.0651
PDGFB	Platelet derived growth factor subunit B	↑	0.0029	0.0651
RGS5	Regulator of G-protein signaling 5	↓	0.0009	0.0651
ERG	ERG, ETS transcription factor	↑	0.0055	0.0869
JAG1	Jagged 1	↓	0.0073	0.0956
IL16	Interleukin 16		0.009	0.1020
TGFB1	Transforming growth factor beta 1	↑	0.0104	0.1024
ANGPTL6	Angiopoietin like 6	↓	0.0118	0.1037
PRDX1	Peroxiredoxin 1	↑	0.0186	0.1468
PLG	Plasminogen	↓	0.022	0.1581
PLAU	Plasminogen activator, urokinase	↑	0.0264	0.1738
UPB1	Beta-ureidopropionase 1	↓	0.0317	0.1925
PECAM1	Platelet and endothelial cell adhesion molecule 1	↓	0.0355	0.2006
HIF1A	Hypoxia inducible factor 1 alpha subunit	↑	0.0453	0.2236
SERPINA7	Serpin family A member 7	↓	0.0443	0.2236

P values and FDRs are indicated (FDR cut off at 0.25).

### Identification of differentially expressed genes in NHP livers with anti-DLL4-induced SD

Hepatic gene expression patterns were also evaluated in entire sections of liver from NHP livers with or without SD following treatment with 5, 15 and 50 mg/kg of anti-DLL4 or vehicle control. The sample set comprised NHP liver samples without SD (vehicle, n = 6, and 5 mg/kg anti-DLL4, n = 1), and with SD (5, 15 or 50 mg/kg anti-DLL4, n = 17). Twenty-six differentially expressed hepatic genes were identified with FDR<0.25, of which 11 genes reached significance (FDR<0.05) (Table 2). PCA separated samples according to the expression profiles of the identified 26 hepatic genes (FDR<0.25) and revealed a total contribution rate of the first 3 principal components that reached 48.73% of the total data variability and partitioned samples into distinct domains of 3-D space based on presence and absence of SD (Fig 2C). The identified gene-set (FDR<0.25) also separated liver samples into 2 clusters based on presence and absence of SD in hierarchical clustering (Fig 2D). Of the significant genes (FDR<0.05), EFNB2 was down-regulated, whereas remaining genes were up-regulated in livers with SD. The NHP liver sample from a 5 mg/kg animal (ID I00694) did not have SD, despite similar drug exposure to others in the dose group (data not shown) and was clearly discriminated from samples with SD by PCA and clustered according to SD status with vehicle group (Fig 2C and 2D). Therefore, hierarchical analysis and PCA of the hepatic gene expression profiles for all animals suggested that the presence of SD could be differentiated in NHPs treated with anti-DLL4.

**Table 2:** List of differentially expressed genes in whole liver samples-derived from NHPs with any anti-DLL4-associated SD (severity score 1–3, SD 1–3) (n = 17) compared to whole liver samples-derived from NHPs without anti-DLL4-associated SD (severity score 0, SD 0) (n = 7).

Gene symbol	Gene name	Direction	p-value (SD 1–3 vs. SD 0)	FDR (SD 1–3 vs. SD 0)
EFNB2	Ephrin B2	↓	0.0002	0.0087
NOTCH4	Notch 4	↑	0.0002	0.0087
LFNG	LFNG O-fucosylpeptide 3-beta-N-acetylglucosaminyltransferase	↑	0.0005	0.0133
ERG	ERG, ETS transcription factor	↑	0.0007	0.0155
NOTCH1	Notch 1	↑	0.0009	0.0155
CCND1	Cyclin D1	↑	0.0016	0.0234
CCL2	C-C motif chemokine ligand 2	↑	0.0022	0.0268
ICAM1	Intercellular adhesion molecule 1	↑	0.0030	0.0291
TGFB1	Transforming growth factor beta 1	↑	0.0029	0.0291
IL16	Interleukin 16	↑	0.0049	0.0401
PRDX1	Peroxiredoxin 1	↑	0.0051	0.0401
MMP9	Matrix metallopeptidase 9	↑	0.0085	0.0604
PDGFB	Platelet derived growth factor subunit B	↑	0.0090	0.0604
PTGS2	Prostaglandin-endoperoxide synthase 2	↑	0.0105	0.0655
CDH5	Cadherin 5	↑	0.0123	0.0667
EGFL7	EGF like domain multiple 7		0.0122	0.0667
HEY1	Hes related family bHLH transcription factor with YRPW motif 1	↓	0.0194	0.0937
STAB2	Stabilin 2	↑	0.0190	0.0937
ANGPTL6	Angiopoietin like 6	↓	0.0366	0.1676
RGS5	Regulator of G-protein signaling 5	↓	0.0475	0.2066
SERPINE1	Serpin family E member 1	↑	0.0515	0.2134
CD163	CD163 molecule	↓	0.0596	0.2141
COX4I1	Cytochrome c oxidase subunit 4I1	↓	0.0593	0.2141
CXCL6	C-X-C motif chemokine ligand 6	↑	0.0608	0.2141
MKI67	Marker of proliferation Ki-67	↑	0.0640	0.2141
NOS3	Nitric oxide synthase 3	↑	0.0627	0.2141

P values and FDRs are indicated (FDR cut off at 0.25).

### Association between bev treatment and SD in CRLM patients

Among 40 patients studied that received oxaliplatin treatment, 13 (32.5%) had moderate or severe (grade 2 or 3) SD, and 27 (67.5%) had either a mild lesion or absence of SD (grade 0 or 1). In contrast, among 25 patients studied that received oxaliplatin and bev treatment, 24 (96%) had either no or mild (grade 0 or 1) SD, and only 1 (4%) patient had severe (grade 3) lesions. In this group, there were no patients who developed moderate SD (Table 3). These differences were statistically significant (P = 0.02). All patients within these respective groups were comparable for age, gender, body mass index and time between the end of treatment and surgery.

**Table 3:** Patient characteristics.

	Oxaliplatin (N = 40)		Oxaliplatin+bev (N = 25)		P-value
	Value	% or IQR	Value	% or IQR	
***Patient Characteristics***					
Male	25	63%	15	60%	0.84
Age (Median)	62	55–67	62	54–70	0.98
*Primary Tumor*					
Rectal cancer	18	45%	8	32%	0.29
T3/T4	26	77%	16	76%	0.98
Lymph node	24	67%	10	48%	0.16
Adjuvant CTx	2	5%	2	8%	0.64
Neoadjuvant Rtx	17	43%	6	24%	0.13
***Liver Metastases***					
Synchronous (≤12 months)	38	95%	23	92%	0.62
Bilobar distribution	24	60%	15	60%	1
Number >1	34	85%	21	84%	0.91
Largest metastasis >5cm	8	21%	7	29%	0.47
CEA>200 ng/ml	6	22%	2	12%	0.47
Clinical risk score 3–5	25	63%	16	67%	0.74
R1 resection	13	33%	6	25%	0.53
***SD***					
Absent	19	48%	21	84%	0.02
Mild	8	20%	3	12%	
Moderate	6	15%	0	0%	
Severe	7	18%	1	4%	

### Identification of differentially expressed genes in patient livers with oxaliplatin-induced SD

**Fig 3:**
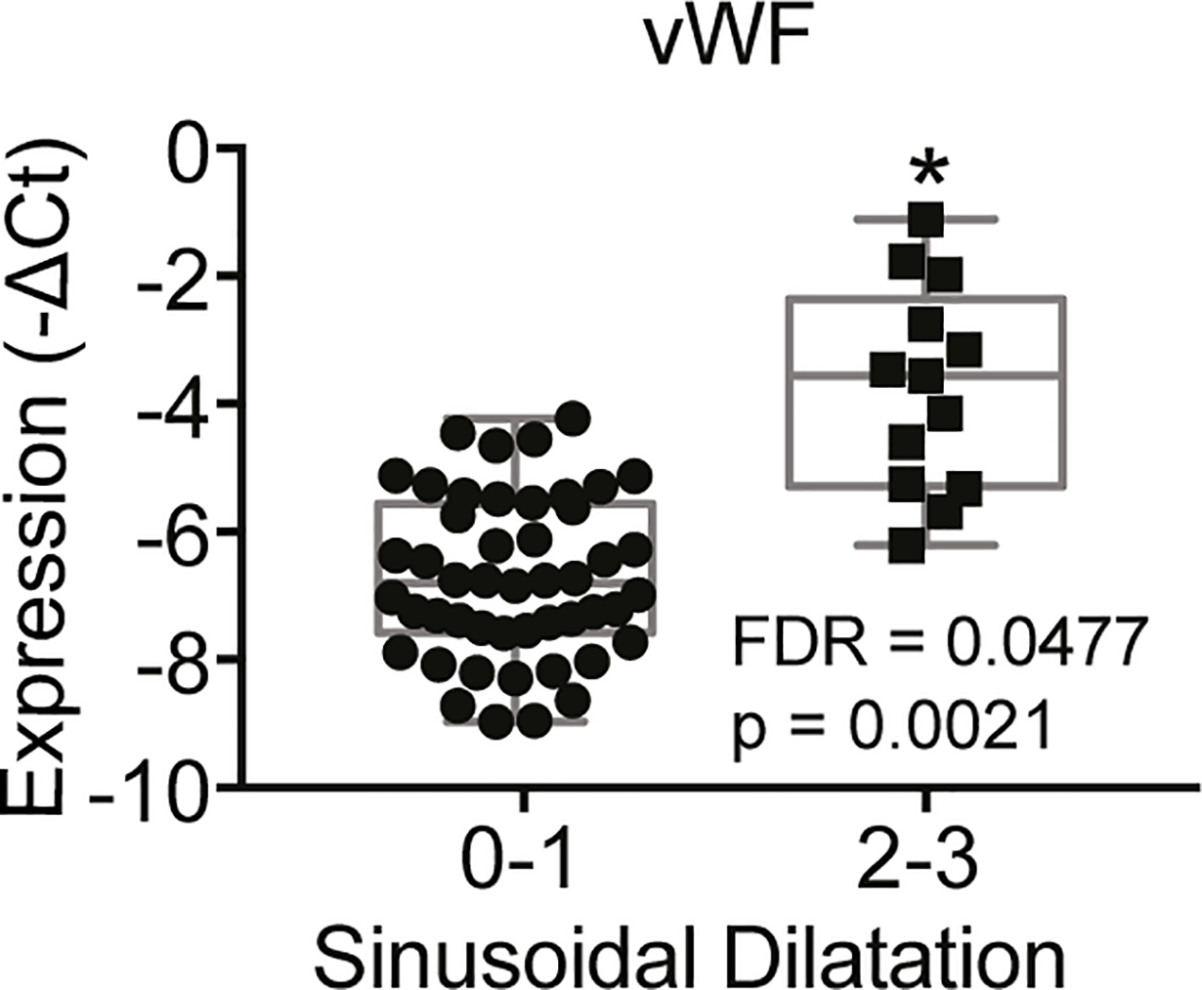
Box-plot indicating gene affected by an overall SD effect among oxaliplatin (+/- bev) treated CRLM patients (SD severity score 0–1 (n = 48) vs. SD severity score 2–3 (n = 13)). Two-way ANOVA with treatment and SD status as factors was used. P value and FDR<0.05 are indicated in each box plot graph. OX = oxaliplatin, Bev = bevacizumab.

To study the overall effect of moderate/severe SD on hepatic gene expression in CRLM patients, a comparison was made between CRLM patients with moderate/severe SD (n = 13) and CRLM patients with absent/mild SD (n = 48). The similarity between microscopic changes in livers of CRLM patients and the NHP model are shown Fig 1. Only VWF expression was significantly up-regulated (FDR = 0.048) (Fig 3).

**Fig 4:**
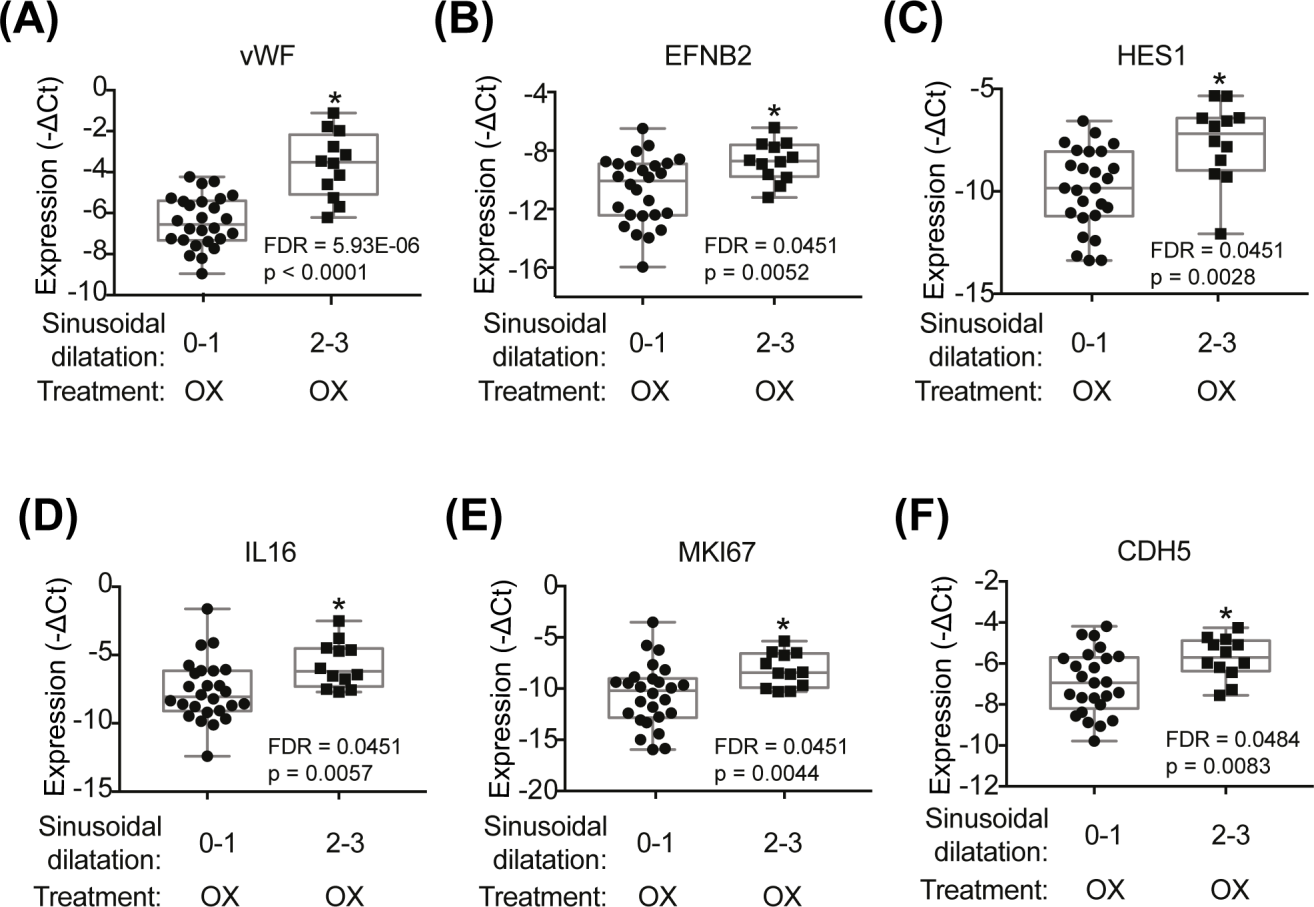
Box-plots indicating genes affected by SD effect among oxaliplatin only-treated CRML patients (SD severity score 0–1 (n = 26) vs. SD severity score 2–3 (n = 12)). Two-way ANOVA with treatment and SD status as factors was used. P value and FDR<0.05 are indicated in each box plot graph **(A-F)**. OX = oxaliplatin, Bev = bevacizumab.

The differential effect of the presence of moderate/severe SD on hepatic gene expression in comparison to absent/mild SD was investigated by controlling for treatment effect in CRLM patients, limiting to subjects with oxaliplatin-only treatment. The sample set included n = 12 CRLM patients with moderate/severe SD and n = 26 CRLM patients with absent/mild SD. Sixteen differentially expressed hepatic genes were identified with FDR<0.25 (Table 4), of which 6 genes reached significance (FDR<0.05) (Fig 4).

**Table 4:** List of differentially expressed genes in CRLM patients with moderate to severe oxaliplatin (+/- bev)-associated SD (severity score 2–3, SD 2–3) compared to CRLM patients without or mild oxaliplatin (+/- bev)-associated SD (severity score 0–1, SD 0–1).

Gene symbol	Gene name	Direction	p-value (SD 2–3 vs. SD 0–1)	FDR (SD 2–3 vs. SD 0–1)
	**Overall comparison (all subjects) (SD 2–3 (n = 13) vs. SD 0–1 (n = 48))**			
VWF	Von Willebrand factor	↑	0.0021	0.0477
	**Comparison limited to subjects with oxaliplatin-only treatment (SD 2–3 (n = 12) vs. SD 0–1 (n = 26))**			
VWF	Von Willebrand factor	↑	<0.0001	5.93E-06
EFNB2	Ephrin B2	↑	0.0052	0.0451
HES1	Hes family bHLH transcription factor 1	↑	0.0028	0.0451
IL16	Interleukin 16	↑	0.0057	0.0451
MKI67	Marker of proliferation Ki-67	↑	0.0044	0.0451
CDH5	Cadherin 5	↑	0.0083	0.0484
CCL2	C-C motif chemokine ligand 2	↑	0.0130	0.0649
PODXL	Podocalyxin like	↑	0.0132	0.0649
MMP2	Matrix metallopeptidase 2	↑	0.0157	0.0718
NOTCH4	Notch 4	↑	0.0257	0.1029
ICAM1	Intercellular adhesion molecule 1	↑	0.0285	0.1074
LFNG	LFNG O-fucosylpeptide 3-beta-N-acetylglucosaminyltransferase	↑	0.0351	0.1248
ERG	ERG, ETS transcription factor	↑	0.0475	0.1601
IL6	Interleukin 6	↑	0.0593	0.1872
JAG1	Jagged 1	↑	0.0614	0.1872
FLT1	Fms related tyrosine kinase 1	↑	0.0732	0.2131

P values and FDRs are indicated (FDR cut off at 0.25).

### Interrogation of the protective mechanisms of bev in oxaliplatin-induced SD

**Fig 5:**
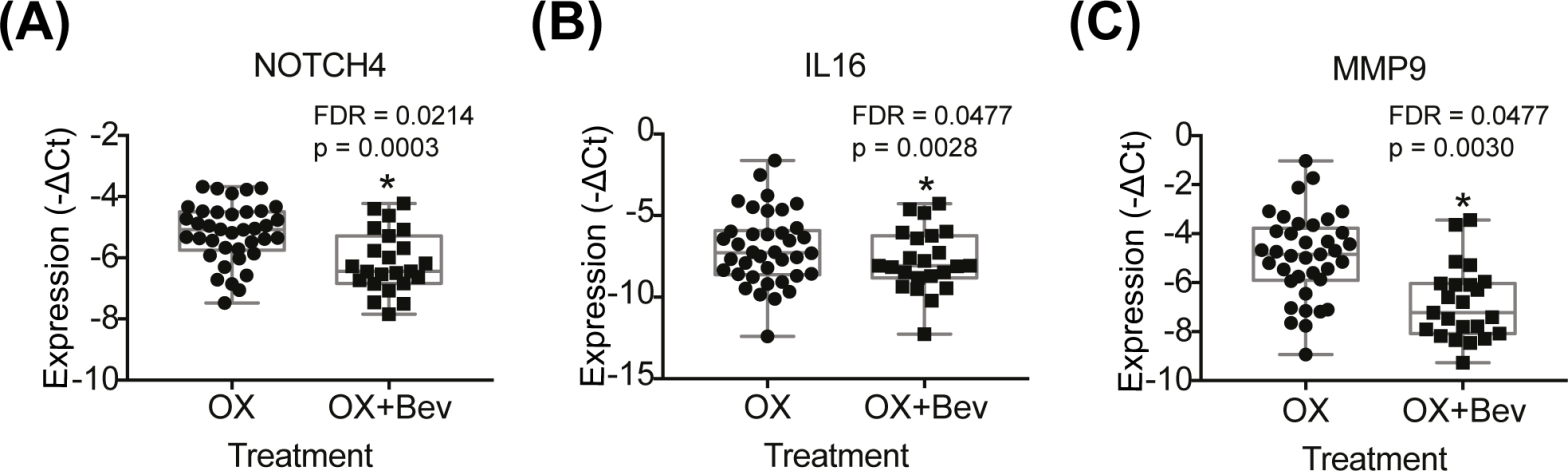
Box-plots indicating genes affected by an overall treatment effect (oxaliplatin (n = 38) vs. oxaliplatin + bev (n = 23)). Two-way ANOVA with treatment and SD status as factors was used. P-value and FDR < 0.05 are indicated in each box plot graph **(A-C)**. OX = oxaliplatin, Bev = bevacizumab.

To study the overall effect of bev addition to an oxaliplatin regimen on hepatic gene expression, a comparison was made between patients that were treated with oxaliplatin and bev (SD severity scores 0–3; n = 23) and patients that were treated with oxaliplatin only (SD severity scores 0–3; n = 38). Seven differentially expressed hepatic genes were identified with FDR<0.25 (Table 5), of which 3 genes reached significance FDR<0.05 (Fig 5).

**Table 5:** List of differentially expressed genes in CRLM patients receiving oxaliplatin and bev treatment compared to CRLM patients receiving oxaliplatin alone treatment.

Gene symbol	Gene name	Direction	p-value (OX + Bev vs. OX)	FDR (OX + Bev vs. OX)
**Overall comparison (all subjects) (OX + Bev (n = 38) vs. OX (n = 23))**				
NOTCH4	Notch 4	↓	0.0003	0.0214
IL16	Interleukin 16	↓	0.0028	0.0477
MMP9	Matrix metallopeptidase 9	↓	0.0030	0.0477
EFNB2	Ephrin B2	↓	0.0117	0.1494
JAG1	Jagged 1	↓	0.0289	0.2313
NOTCH1	Notch 1	↓	0.0278	0.2313
PODXL	Podocalyxin like	↓	0.0257	0.2313
**Comparison limited to subjects with no or mild SD (OX (n = 26) + Bev vs. OX (n = 22))**				
CDH5	Cadherin 5	↑	0.0046	0.0451
HEY1	Hes related family bHLH transcription factor with YRPW motif 1	↓	0.0068	0.0451
JAG1	Jagged 1	↓	0.0036	0.0451
MMP9	Matrix metallopeptidase 9	↓	0.0018	0.0451
TIMP1	TIMP metallopeptidase inhibitor 1	↓	0.0070	0.0451
NOTCH4	Notch 4	↓	0.0184	0.0784
VWF	Von Willebrand factor	↓	0.0822	0.2287

P values and FDRs are indicated (FDR cut off at 0.25).

**Fig 6:**
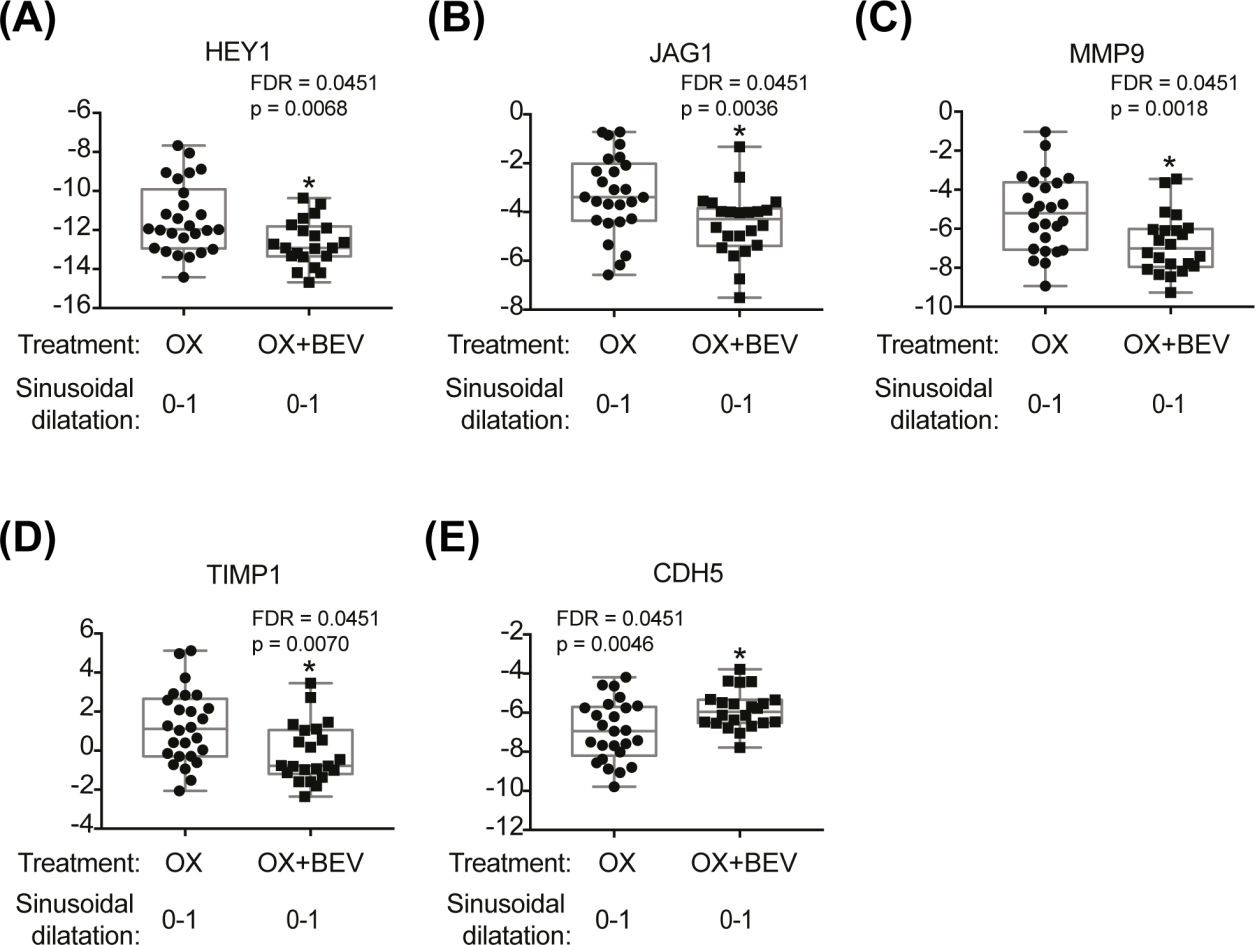
Box-plots indicating genes affected by treatment effect among CRML patients with no or mild SD only (oxaliplatin (n = 26) vs. oxaliplatin + bev (n = 22)). Two-way ANOVA with treatment and SD status as factors was used. P-value and FDR < 0.05 are indicated in each box plot graph **(A-E)**. OX = oxaliplatin, Bev = bevacizumab.

The differential effect of bev on hepatic gene expression in comparison to oxaliplatin was investigated by controlling for liver SD, limiting to subjects with absent/mild SD. The sample set included 22 CRLM patients treated with oxaliplatin and bev and 26 CRLM patients treated with oxaliplatin only. Seven differentially expressed hepatic genes were identified with a FDR<0.25 (Table 5), of which 5 genes reached significance (FDR<0.05) (Fig 6).

## Discussion

To date, several lines of evidence have implicated NOTCH dysregulation in a variety of pharmacologic, genetic, and toxicologic models of SD [20–22]. In the current study, we investigated transcriptomic changes associated with anti-cancer drug-induced hepatic SD. We reasoned that the development of this uncommon hepatotoxicity following treatment with either oxaliplatin or anti-DLL4 was related to a similar pathological mechanism involving NOTCH signaling. Here, we have employed a NHP model of anti-DLL4 induced SD, which reliably recapitulates the clinical pathology [12, 15]. We have further investigated the molecular pathogenesis oxaliplatin-induced SD in CRLM patient samples. The protective effect of bev against oxaliplatin-induced SD was interrogated by comparing cases from patients who received oxaliplatin treatment with or without anti-VEGF therapy. Using robust targeted transcriptomics analyses we observed important overlap in the genes altered in both the NHP anti-DLL4- and CRLM oxaliplatin-induced SD models. Genes with expression changes common to both anti-DLL4 and oxaliplatin contexts included EFNB2 (downregulated and upregulated respectively) and IL16 (upregulated in both settings) FDR < 0.05), with CCL2, ERG, ICAM1, LFNG and NOTCH4 (upregulated in both settings; FDR<0.05 in NHP and < 0.25 in oxaliplatin-SD). We also observed significantly higher CDH5 and lower HEY1, IL16, JAG1, MMP9, NOTCH4 and TIMP1 in bev-treated patients ((FDR<0.05). Notably, CDH5, IL16, MMP9, NOTCH4 (upregulated) and HEY1 (down-regulated) (FDR<0.25) were also implicated in the anti-DLL4-induced SD NHP model. Collectively, these findings support the hypothesis that NOTCH signaling pathway plays an important role in the maintenance of sinusoidal homeostasis and further implicates NOTCH in the pathogenesis of anti-cancer drug-induced SD. Moreover, IL16 is identified as a putative novel disease marker.

Four NOTCH receptors (NOTCH1 to NOTCH4) and trans-membrane ligands in the Delta-like (DLL1 to DLL4) and Jagged (JAG1 and JAG2) protein families comprise the core components of the NOTCH pathway which influences many developmental and homeostatic processes [23]. NOTCH signaling can regulate endothelial cell homeostasis, however to date, it has not been directly associated with drug-induced SD [24]. Over-expression of active NOTCH4 results in abnormally dilated hepatic vascular structures [25]. Similarly, inhibition of NOTCH1 has been shown to cause disturbance of hepatic sinusoidal vascular homeostasis implicating the NOTCH signaling pathway in maintenance of a quiescent liver endothelium [12, 20]. A direct NOTCH target, EFNB2 receptor tyrosine kinase is involved in endothelial cell angiogenesis and the regulation of vessel morphology [26]. Moreover, it has previously been reported that deletion of EFNB2 results in vascular remodeling defects [27]. We observed significant down-regulation of EFNB2 (FDR<0.05) in our NHP model. Down-regulation of this gene was previously observed with down-regulation of DLL4 in liver sinusoidal endothelial cells (LSECs) isolated from constitutive NOTCH1 knockout mice. Patients with nodular regenerative hyperplasia further display involvement of DLL4/NOTCH1 and EFNB2/EPHB4 pathways in hepatic vascular homeostasis and remodeling [21]. Interestingly, we observed up-regulation of EFNB2 in CRLM patients with oxaliplatin-associated SD. In this context, the increase of EFNB2 expression could be driven by NOTCH4, as shown *in vitro* with pre-eclamptic endothelial progenitor cells [28]. Therefore, disruption of EFNB2 homeostasis in either direction (increase or decrease) could potentially disturb vascular homeostasis and result in SD. Alternatively, as perisinusoidal fibrosis was evident as a feature of oxaliplatin-induced SD in our human case series [13] and not in anti-DLL4 SD [12, 15, 16], increased EFNB2 expression could be related to active fibrogenesis. Involvement of EphB2 receptor in liver fibrosis has been demonstrated in mouse models where expression was upregulated in activated hepatic stellate cells which promoted fibrogenesis [29].

Previous studies have further proposed an association between the NOTCH pathway and inflammation [22, 30]. Indeed, in both drug–induced SD models presented, we have observed an up-regulation of genes involved in inflammation and repair, including IL16 (FDR<0.05 in both models) and CCL2 (FDR<0.05 in anti-DLL4 model and with higher FDR in oxaliplatin model). Up-regulation of TGFB1 was also evident in our anti-DLl4-induced SD NHP model (FDR<0.05). IL16 is an immunomodulatory cytokine that is a chemoattractant for CD4+ immune cells [31] though not previously associated with drug-induced SD. Recently, novel physiological functions of IL16 in vascular smooth muscle cells, including IL16-induced MMP9 expression, have been described [32]. IL16 has also been shown to be an inflammatory mediator involved in cardiotoxicity; increased IL16 expression in the heart of transgenic mice was shown to result in cardiac fibrosis, left ventricle myocardial stiffening, and increased macrophage infiltration [33]. CCL2, on the other hand, is NOTCH pathway dependent [30]. Shen et al. have recently demonstrated the importance of DLL4 in liver disease and its role in the modulation of liver inflammatory response by down-regulation of CCL2 chemokine expression [30]. A trend towards up-regulation of CCL2 in CRLM patients with oxaliplatin-induced SD (FDR<0.25) and up-regulation of CCL2 in NHPs with anti-DLL4-induced SD (FDR<0.05), further suggest involvement of the NOTCH pathway in the pathogenesis of drug-induced SD. Finally, CCL2 has previously been shown to be up-regulated in CRLM patients with oxaliplatin-induced SD [34].

Interestingly, we have further observed increased ERG expression in anti-DLL4 induced SD (FDR<0.05) with trend towards up-regulation in CRLM patients with oxaliplatin-induced SD (FDR<0.25). ERG has been implicated in vascular development and angiogenesis and maintenance of vascular quiescence and stability. ERG has also been shown to control Wnt/B-catenin pathway by driving expression VE-cadherin (CDH5), an adhesion molecule that controls the integrity of endothelial junctions [35]. Consistent with this pathway, we observed CDH5 up-regulation in oxaliplatin-induced SD. Perturbations in expression of a marker of vascular integrity (ICAM1) and a cell cycle regulatory gene (CCND1) were also observed and may further indicate loss of vascular homeostasis. In agreement with previous reports, our study further underlines the importance of vWF in oxaliplatin-induced SD [6, 34].

By studying bev’s protective effect against oxaliplatin-induced SD, we further confirmed the importance of the NOTCH signaling pathway in SD pathogenesis. A subset of patients used in these analyses were previously included in a larger published study by van der Pool *et al*. that noted a similar amelioration of SD attributed to bev [13]. Our smaller sample set is also consistent with a larger CRLM patient cohort where Rubbia-Brandt *et al*. provided a detailed assessment of hepatic lesions associated with oxaliplatin-induced hepatotoxicity [7]. We observed down-regulation of NOTCH4, HEY1, JAG1 (all NOTCH related) (FDR<0.05) and a trend towards down-regulation of NOTCH1 (FDR<0.25) in CRLM patients treated with bev and oxaliplatin. The addition of bev to oxaliplatin treatment also resulted in down-regulated MMP9 (FDR<0.05). Activation of matrix metalloproteinases (MMP2 and MMP9) was shown to be involved in the pathogenesis of SD following exposure to monocrotaline (a pyrrolizidine alkaloid) [36] and oxaliplatin [34]. A trend towards increased MMP9 expression was also observed in NHPs with anti-DLL4-induced SD and MMP2 in CRLM patients with oxaliplatin-induced SD (FDR<0.25).

**Fig 7:**
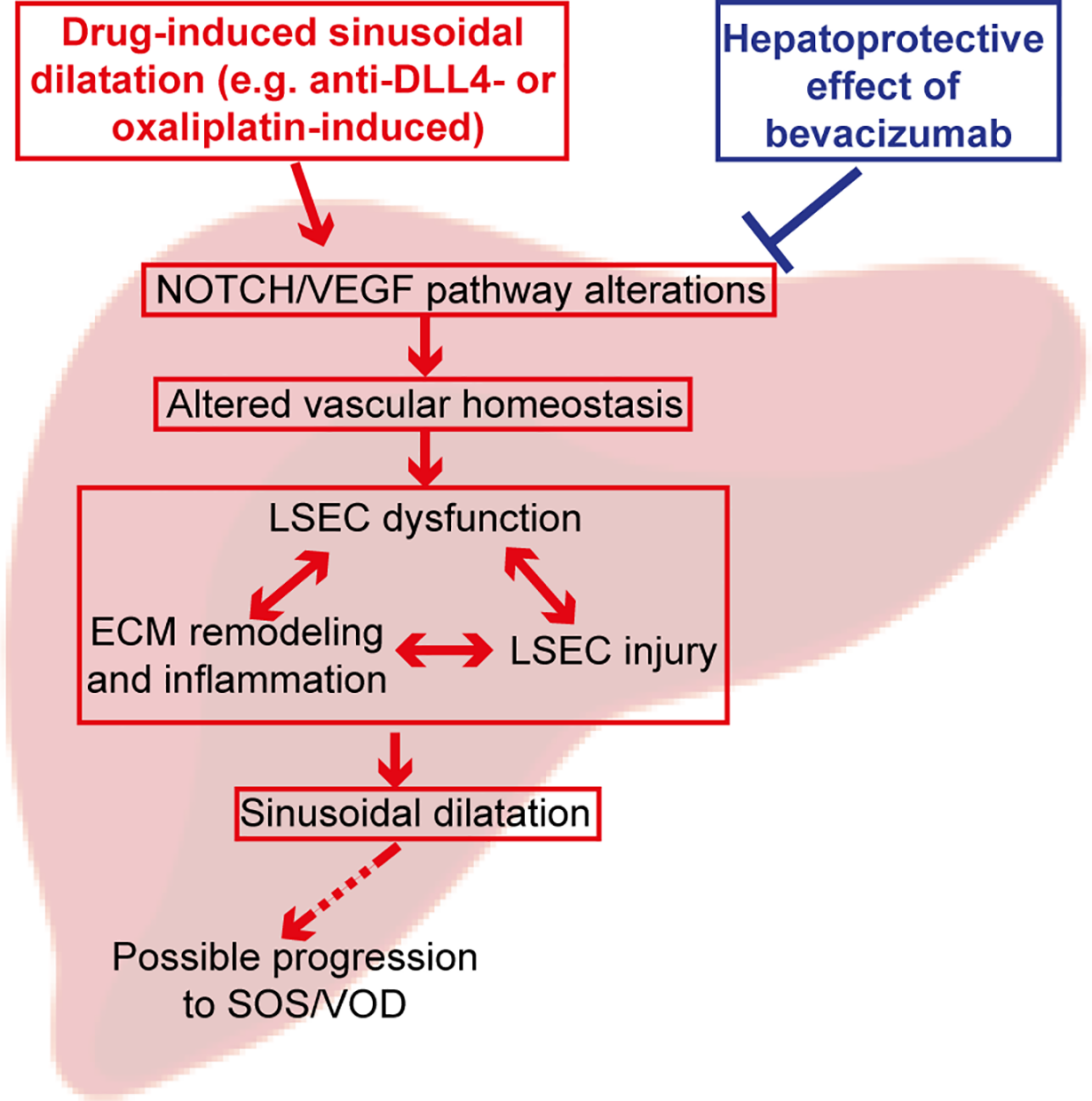
Schematic summary of findings on drug-induced SD based on current analyses in anti-DLL4-induced SD in NHP model and oxaliplatin +/- bev in CRLM patients. Anti-DLL4 and oxaliplatin can lead to dysregulation of NOTCH/VEGF signaling pathways genes in the LSECs. These changes in hepatic gene expression lead to alterations in vascular homeostasis and subsequently to LSEC dysfunction. LSEC dysfunction leads to ECM remodeling, inflammation and eventually to LSEC injury. In response to this SD is developed and may progress to SOS/VOD. Bev hepato-protective effect against OX-induced SD acts through inhibition of NOTCH/VEGF signaling pathways that leads to inhibition of vascular remodeling and inflammation.

Our study provides novel evidence that NOTCH signaling pathway plays a key role in anti-cancer drug-induced SD. We demonstrated that both drugs (anti-DLL4 and oxaliplatin) lead to NOTCH/VEGF pathway perturbations and alter vascular homeostasis. As a consequence, LSECs dysfunction, ECM remodeling, inflammation and LSECs injury collectively lead to SD that may progress to SOS/VOD (Fig 7). Expression of genes involved in the protective effect of bev further confirms our hypothesis that the NOTCH pathway is primarily involved in SD pathogenesis.

### Study limitations and future directions

Regrettably for several reasons it has not been possible to conduct causal studies and robust follow-up immunohistochemistry (IHC) or immunofluorescence (IF) analyses in NHP or patient samples. Specifically, the clinical cohort assessed was not a matched set of pre- and post-chemotherapy biopsies, but rather a cross-sectional study of consecutive material from 65 clinical cases collected between 2009 and 2012 [13]. Further limitations were underpinned by a combination of sample exhaustion, variable sample integrity and biomarker epitope stability. Moreover, while IF studies were attempted in archived NHP samples (inconclusive data not shown) these efforts were beset with difficulties including variability in staining across a section (independent of pathology and expected anatomic localization of the marker). These problems were further complicated by high background signal resulting in the loss of IF detection sensitivity. This phenomenon has previously been observed by others; for example Agostini et al showed up-regulation of thymidylate synthetase RNA in sinusoidal dilation liver cases but did not show a difference in protein levels employing IHC [6]. Future studies to confirm mechanisms of drug-induced SD pathogenesis will benefit from larger sets of recently collected case material to facilitate validation of key candidate targets (IL16, NOTCH1, NOTCH4, JAGGED1, CCL2, ERG, MMP9 and EFNB2) using robust proteomic approaches including IF and/or western blotting.

Notwithstanding these limitations, we nevertheless posit that our data provides important clues as to the molecular events involved in CRC drug-induced SD which are important not only in the ambit of standard of care chemotherapy-induced toxicities, but may also be relevant in the emerging field of anti-DLL4 cancer immunotherapies. For example, monoclonal antibodies against NOTCH have been shown to reduce the cancer stem cell (CSC) population in CRC tumors [37], with clinical trials of therapeutic antibodies against DLL4 in combination with FOLFIRI (irinotecan, folic acid, leucovorin, and fluorouracil), ongoing in the metastatic CRC setting (NCT01189942). Another clinical trial employs the anti-DLL4 antibody demcizumab, in combination with the anti-PD-1 antibody pembrolizumab (immune checkpoint inhibitor) in metastatic solid tumors (NCT02722954) [38]. Understanding pathophysiologic mechanisms of potential hepato-toxicities in these settings thus continues to hold significant clinical relevance.

In conclusion, our data contributes to an improved understanding of the pathophysiologic molecular mechanisms underpinning drug induced SD and SOS/VOD. Going forward, these data may ultimately impact patient care by providing insight into key mechanistic pathways, aid the discovery of safety biomarkers for drug-induced SD and support the development of improved ‘precision’ therapeutics for application in CRC and other tumours. These refined agents would better target specific therapeutic pathways of cancer vulnerability, thereby reducing the incidence and severity of SOS/VOD without reducing drug efficacy. Ultimately, surgical morbidity after hepatic metastasectomy may thus be reduced.

## Supporting information

S1 Fig LCM of NHP liver tissues. NHP liver FFPE sections were stained with ARCTURUS® Paradise® PLUS FFPE LCM Staining Kit and dried before LCM capture. The SD-affected and non-affected hepatic regions (zone 2–3) were identified, cut with laser and captured in caps of PCR test tube. Representative images of each step of LCM for hepatic tissue without SD (SD severity score 0) and with SD (SD severity score 2) are presented (scale bar = 400 μm, top row; or 200 μm, bottom two rows).
(EPS)

S2 Fig Development of a high-throughput microfluidic TaqMan gene expression assay for analysis of FFPE hepatic samples derived from NHPs. Representative six-point standard curves of NHP (rhesus and cynomolgus monkey) normal tissues universal RNA (uRNA) (slope and R-squared values are indicated). Mean ± S.E.M. of technical replicates (n = 3) are presented (A-C).
(TIF)

S3 Fig Development of a high-throughput microfluidic TaqMan gene expression assay for analysis of FFPE hepatic samples derived from NHPs. Representative Ct values of cynomolgus monkey liver total RNA (tRNA), rhesus monkey liver tRNA, human liver tRNA and cynomolgus monkey liver genomic DNA (gDNA) (all at 100 ng input) and no template control (NTC). Mean ± S.E.M. of technical replicates (n = 3) are presented (A-C).
(TIF)

S4 Fig Development of a high-throughput microfluidic TaqMan gene expression assay for analysis of FFPE hepatic samples derived from NHPs. Representative Ct values of RNA extracted from FFPE cynomolgus monkey liver samples that underwent LCM (different captured areas 1, 2, 4, 10 mm2) and RNA extracted from FFPE cynomolgus monkey total liver samples (no LCM). Mean ± S.E.M. of biological replicates (n = 2–4) are presented (A-C).
(TIF)

S5 Fig Development of a high-throughput microfluidic TaqMan gene expression assay for analysis of FFPE hepatic samples derived from NHPs. Intra-chip reproducibility of NHP (cynomolgus and rhesus monkey) uRNA samples (100 ng input) run on the same 96.96 Dynamic Array (R-squared values are indicated). Mean of technical replicates (n = 3) are presented (A-C). Inter-chip reproducibility of the TaqMan gene expression assay assessed by comparing Ct values of NHP (cynomolgus and rhesus monkey) uRNA (100 ng input) across three independent assay runs using three different 96.96 Dynamic Arrays (R-squared values are indicated). Mean of technical replicates (n = 3) are presented (D-F).
(TIF)

S1 File Supplementary material. (DOCX)

S2 File NC3Rs ARRIVE guidelines checklist. (PDF)

S3 File Raw fluidigm files. (ZIP)
