# Supplementary figures and images for: Interrogation of transcriptomic changes associated with drug-induced hepatic sinusoidal dilatation in colorectal cancer
Source: PLoS One. 2018 Jun 7;13(6):e0198099. doi: 10.1371/journal.pone.0198099 (PMC5991753; doi:10.1371/journal.pone.0198099)

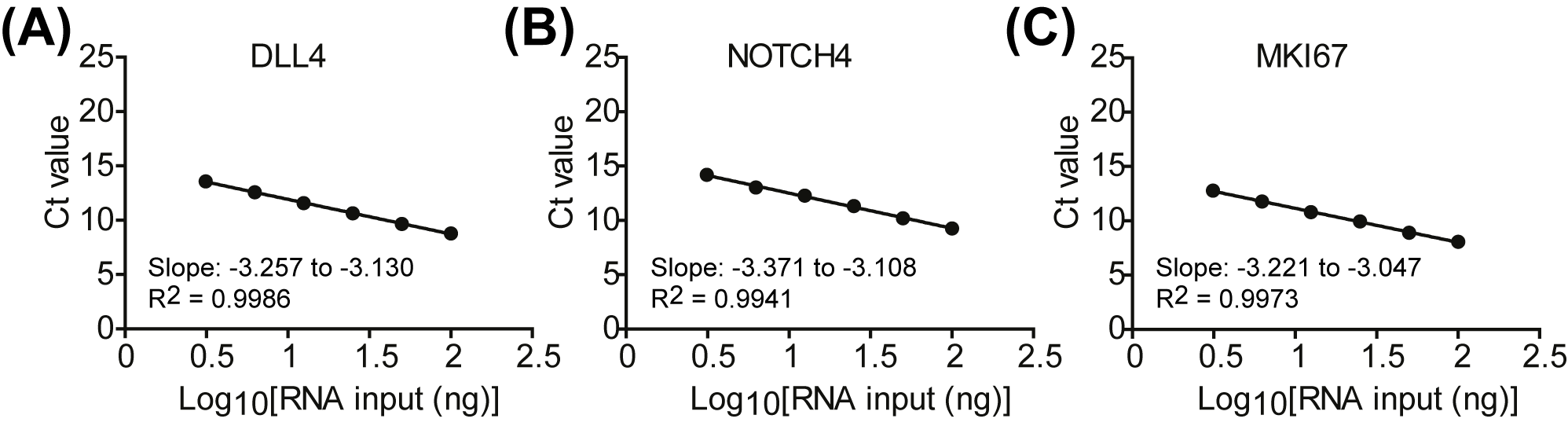

Supplement: S2 Fig — Representative six-point standard curves of NHP (rhesus and cynomolgus monkey) normal tissues universal RNA (uRNA) (slope and R-squared values are indicated). Mean ± S.E.M. of technical replicates (n = 3) are presented (A-C). (TIF) [file pone.0198099.s002.tif]

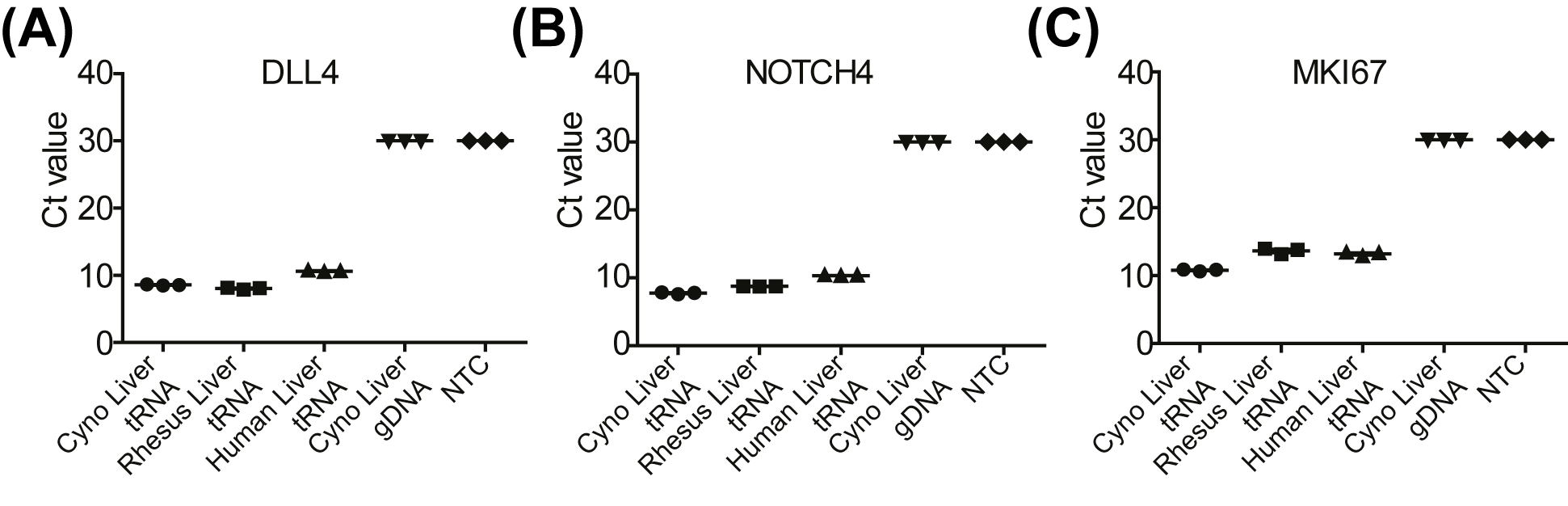

Supplement: S3 Fig — Representative Ct values of cynomolgus monkey liver total RNA (tRNA), rhesus monkey liver tRNA, human liver tRNA and cynomolgus monkey liver genomic DNA (gDNA) (all at 100 ng input) and no template control (NTC). Mean ± S.E.M. of technical replicates (n = 3) are presented (A-C). (TIF) [file pone.0198099.s003.tif]

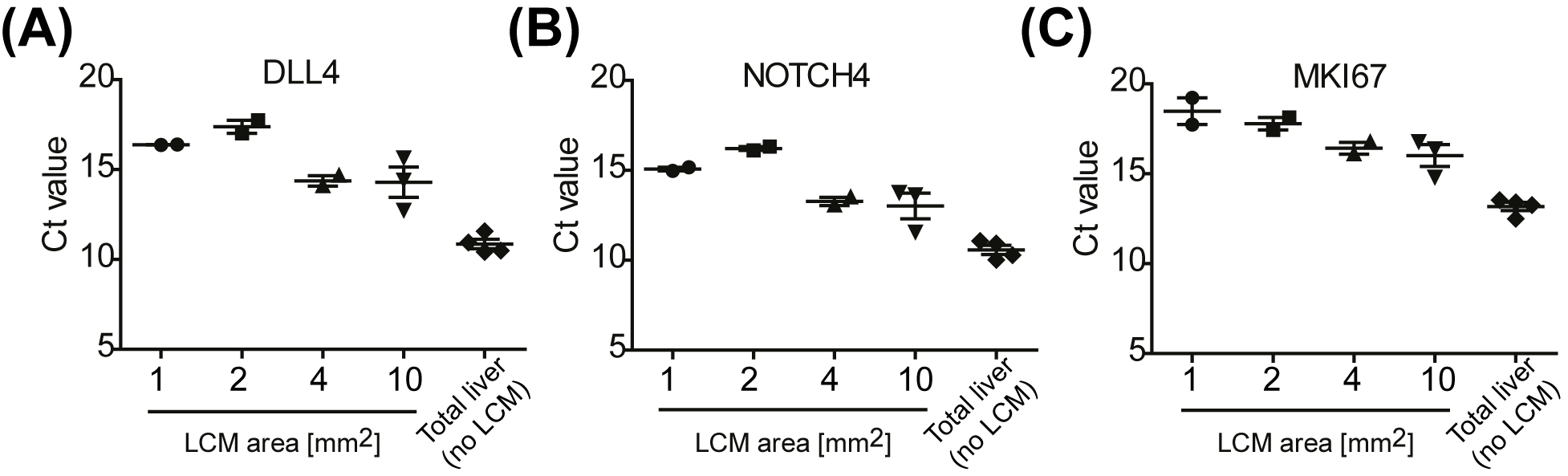

Supplement: S4 Fig — Representative Ct values of RNA extracted from FFPE cynomolgus monkey liver samples that underwent LCM (different captured areas 1, 2, 4, 10 mm2) and RNA extracted from FFPE cynomolgus monkey total liver samples (no LCM). Mean ± S.E.M. of biological replicates (n = 2–4) are presented (A-C). (TIF) [file pone.0198099.s004.tif]

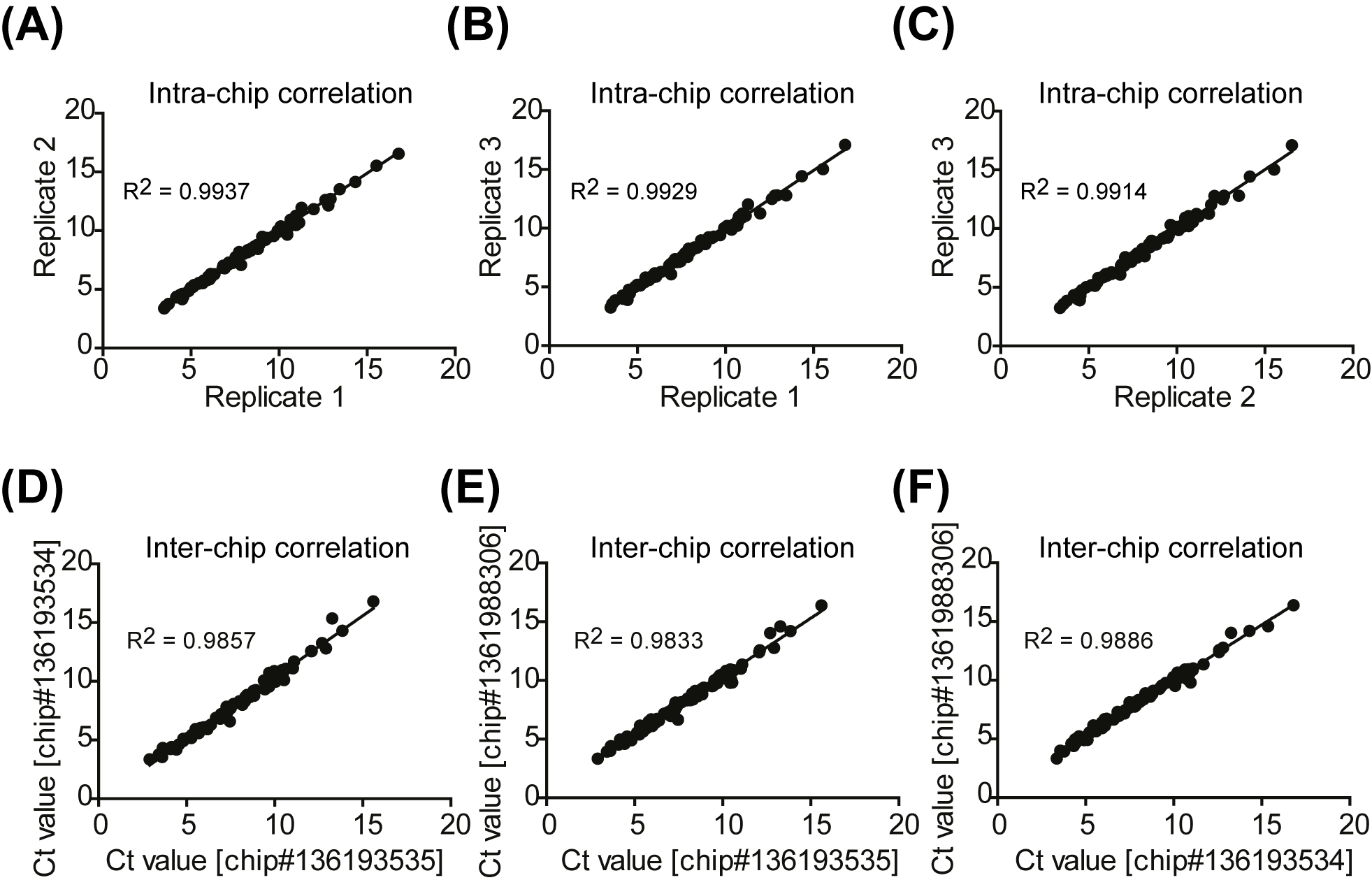

Supplement: S5 Fig — Intra-chip reproducibility of NHP (cynomolgus and rhesus monkey) uRNA samples (100 ng input) run on the same 96.96 Dynamic Array (R-squared values are indicated). Mean of technical replicates (n = 3) are presented (A-C). Inter-chip reproducibility of the TaqMan gene expression assay assessed by comparing Ct values of NHP (cynomolgus and rhesus monkey) uRNA (100 ng input) across three independent assay runs using three different 96.96 Dynamic Arrays (R-squared values are indicated). Mean of technical replicates (n = 3) are presented (D-F). (TIF) [file pone.0198099.s005.tif]
