# Supplementary material for: Interrogation of transcriptomic changes associated with drug-induced hepatic sinusoidal dilatation in colorectal cancer
Source: PLoS One. 2018 Jun 7;13(6):e0198099. doi: 10.1371/journal.pone.0198099 (PMC5991753; doi:10.1371/journal.pone.0198099)
Supplement: S1 File — (DOCX) [file pone.0198099.s006.docx]

**Supplementary Materials and Methods**

**High-throughput real-time quantitative PCR of NHP and clinical samples**

Prior to loading the samples and assays into the inlets, the 96.96 Dynamic Array integrated fluidic circuits (IFC) (Fluidigm, South San Francisco, CA) was primed in the IFC Controller. 20x TaqMan gene expression assays were 1:2 diluted using the Assay Loading Reagent (Fluidigm, South San Francisco, CA). Pre-amplified cDNA samples were mixed with 2x TaqMan Universal PCR Mix (Applied Biosystems, Foster City, CA) and Sample Loading Reagent (Fluidigm, South San Francisco, CA) (1:1:1:0.1 dilution). Five µl of 10x TaqMan gene expression assay mix was then loaded into each detector inlet of the dynamic array chip and five µl of pre-amplified cDNA sample prepared as described above was loaded into each sample inlet in triplicates. The loaded chip was then placed into IFC Controller for loading and mixing. Subsequently, the chip was transferred to the BioMark^TM^ Real-Time PCR System. The cycling program used consisted of 2 min at 50°C, 30 min at 70°C, 10 min at 25°C, 2 min at 50°C and 10 min at 95°C (1 cycle each) followed by 40 cycles of 95°C for 15 sec and 1 min at 60°C. Ct values were obtained using Fluidigm RT-qPCR Analysis software (Fluidigm, South San Francisco, CA). The quality threshold was set at the default setting of 0.65. Derivative baseline correction method, automatic threshold for each assay and quality threshold set at 0.65 were used. Non-template, universal and total RNA controls were included on each chip. Samples/assays with majority of lower level of detection (LLOD) values were excluded from further analysis. Three technical replicates were compared and Ct difference equal 2 was used as a threshold to exclude technical outliers.

**Validation of TaqMan gene expression assays for species and tissue specificity**

In order to characterize the molecular pathology of anti-DLL4-induced hepatic SD in NHP livers, TaqMan gene expression assays were validated using high-throughput, microfluidic RT-qPCR for the simultaneous analysis of up to 96 multiple genes. The TaqMan gene expression assay panel content was selected to best represent key genes involved in VEGF/NOTCH pathway, molecular markers associated with different models of drug-induced SD, and liver and vascular toxicity markers based on a review of literature for genes implicated in sinusoidal dilatation, sinusoidal obstruction syndrome, and NOTCH signaling in vascular homeostasis. Also included were genes from a “tumor angiogenesis” panel previously validated by Genentech, Inc. on clinical samples for tumor angiogenesis drugs including bevacizumab (1-3). Each assay was individually validated for linearity and sensitivity by testing on a range of RNA inputs (3.13-100 ng (1:2 dilutions)) from NHP (rhesus and cynomolgus monkey) normal tissues universal RNA (uRNA) **(Figure S2)**. Cynomolgus monkey liver total RNA (tRNA), rhesus monkey liver tRNA, human liver tRNA and cynomolgus monkey liver genomic DNA (gDNA) (all at 100 ng input) and no template control (NTC) were used in triplicates to determine TaqMan gene expression assay specificity to cynomongus monkey liver **(Figure S3)**. Sensitivity of TaqMan gene expression assays was also determined using RNA isolated either from total liver or from 1, 2, 4 or 10 mm^2^ LCM hepatic regions subjected to pre-amplification with gene specific primers **(Figure S4)**. To further validate the TaqMan gene expression assay panel, NHP (rhesus and cynomolgus monkey) normal tissues uRNA (100 ng input) were run in triplicate on the same chip and found to show strong intra-chip reproducibility (R^2^ > 0.99) **(Figure S5A-C)**. NHP (rhesus and cynomolgus monkey) normal tissues uRNA (100 ng input) Ct values across three independent chips were also compared and showed strong inter-chip reproducibility (R^2^ > 0.98) (**Figure S5D-F**).

**References**

1. BRAUER M J, ZHUANG G, SCHMIDT M, et al. Identification and analysis of in vivo VEGF downstream markers link VEGF pathway activity with efficacy of anti-VEGF therapies. Clin Cancer Res 2013; 19(13): 3681-92.

2. WALLIN J J, BENDELL J C, FUNKE R, et al. Atezolizumab in combination with bevacizumab enhances antigen-specific T-cell migration in metastatic renal cell carcinoma. Nat Commun 2016; 7: 12624.

3. GARCIA-CARBONERO R, VAN CUTSEM E, RIVERA F, et al. Randomized Phase II Trial of Parsatuzumab (Anti-EGFL7) or Placebo in Combination with FOLFOX and Bevacizumab for First-Line Metastatic Colorectal Cancer. Oncologist 2017; 22(4): 375-e30.
